# Views and Experiences of New Zealand Women with Gestational Diabetes in Achieving Glycaemic Control Targets: The Views Study

**DOI:** 10.1155/2017/2190812

**Published:** 2017-10-31

**Authors:** Ruth Martis, Julie Brown, Caroline A. Crowther

**Affiliations:** Liggins Institute, The University of Auckland, Private Bag 92019, Auckland 1142, New Zealand

## Abstract

**Introduction:**

Optimal glycaemic control in women with gestational diabetes mellitus (GDM) reduces maternal and infant morbidity.

**Method:**

A survey was administered to women diagnosed with GDM to explore their views and experiences in achieving optimal glycaemic control.

**Results:**

Sixty women participated. Enablers included being taught to test capillary blood glucose in group settings where the health professional demonstrated this on themselves first (60, 100%); health professionals listening (41, 68%); being reminded to perform blood glucose testing (33, 55%); and being provided healthy meals by friends and family (28, 47%). Barriers included not having information in a woman's first language (33, 55%); being offered unhealthy food (19, 31%); not being believed by health professionals (13, 21%); receiving inconsistent information by health professionals (10, 16%); never being seen twice by the same health professional (8, 13%); and long waiting hours at clinics (7, 11%). Two-thirds of women (37, 62%) reported that food costs were not a barrier, but that they were always or frequently hungry.

**Conclusion:**

Optimising experiences for women with GDM for achieving glycaemic control and overcoming barriers, regardless of glycaemic targets, requires further focus on providing meaningful health literacy and support from health professionals, family, friends, and work colleagues.

## 1. Introduction

Globally, there are increasing rates of diabetes, including gestational diabetes mellitus (GDM) [1, 2]. The prevalence of GDM varies among populations but probably affects 10–25% of pregnancies [3–5].

Short- and long-term health risks for women with GDM include preeclampsia, induction of labour, caesarean section, and postnatal depression for the women [6–8]. For the baby, health risks include shoulder dystocia, nerve palsy, preterm birth, neonatal hypoglycaemia, respiratory distress syndrome, and the risk of developing obesity and type 2 diabetes (T2DM) in childhood [9–11].

Treatments for women with GDM that maintain glycaemic control within specified targets have a significant impact on short- and long-term health for the woman and her baby [12–14]. Treatments for GDM include dietary and exercise advice alone or combined with pharmacological therapy [15–19].

While some published studies have described women's experiences of developing gestational diabetes [20–25], little is known as to how women feel about achieving their glycaemic treatment targets. This nested study within the TARGET trial (Australian New Zealand Trial Registry: ACTRN12615000282583) aimed to explore women's views and experiences in achieving their recommended glycaemic treatment targets and to identify potential barriers and enablers.

## 2. Materials and Methods

### 2.1. Participant Selection

Women diagnosed with GDM were eligible to participate if they had a singleton pregnancy, could communicate in English, had been self-monitoring their capillary blood glucose levels for at least two weeks, and provided written consent. Eligible women were sent an email invitation that included a participant information sheet and consent form. Women could choose to be interviewed face to face, or to be telephoned. Women were aware that the survey was not an assessment of their knowledge about GDM and advised that all their information would be kept confidential.

Hospital sites from two different geographical locations in New Zealand participated. Twenty women, recruited from Canterbury District Health Board (DHB) in the South Island, were using less tight glycaemic treatment targets (fasting blood glucose < 5.5 mmol/L, 1 hour postprandial < 8.0 mmol/L, and 2 hours postprandial < 7.0 mmol/L). Forty women were using tighter glycaemic treatment targets (fasting blood glucose ≤ 5.0 mmol/L, 1 hour postprandial ≤ 7.4 mmol/L, and 2 hours postprandial ≤ 6.7 mmol/L): twenty women recruited from Canterbury DHB in the South Island and twenty women recruited from Counties Manukau DHB in the North Island.

Local hospital policies differed for testing of capillary blood glucose. Canterbury DHB moved from initially less tight targets to tighter glycaemic treatment targets during the survey time. Women were asked to test their capillary blood glucose at one hour postprandial. Counties Manukau DHB was using tighter glycaemic treatment targets during the survey time. Women were asked to test their capillary blood glucose two hours postprandial.

### 2.2. The Survey

The survey comprised 45 questions. Twenty questions identified participant demographics and twenty-five their views and knowledge of their glycaemic treatment targets. Questions included identifying what had been helpful in learning how to self-monitor blood glucose levels; support received from family, friends, and health professionals; access to written information; costs associated with their GDM management and treatment; and experience of hunger. The survey was piloted with three women following which three questions were modified. There was an opportunity for women to provide additional information. All women answered all the survey questions.

### 2.3. Analysis

Data analysis was conducted using Pivot Tables in Microsoft Office Excel 2016 calculating frequency and corresponding percentage to describe the responses to the survey questions and included mean and standard deviation for normally distributed data. All analyses were undertaken in Microsoft Office Excel 2016, reporting descriptive statistics for baseline demographics and using simple numeric calculations for survey responses.

### 2.4. Ethical Approval

The Views Survey was nested within the TARGET trial approved by the New Zealand Health and Disability Ethics committee (HDEC) Ref. 14/NTA/163 and research registration number 1965.

## 3. Results

### 3.1. Participants

**Figure 1:**
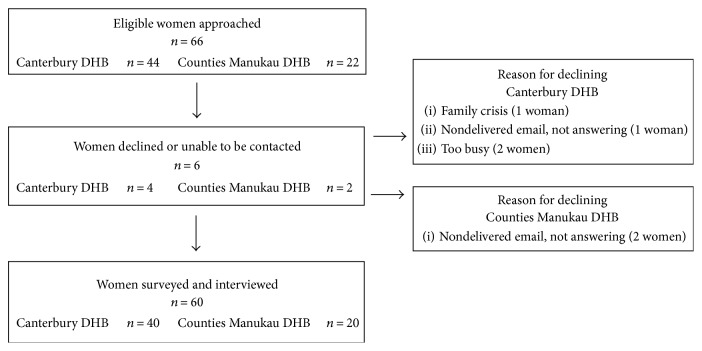
Flowchart of recruitment.

Sixty-six eligible women were approached and sixty women consented to participate in the survey. Six women did not participate because they were too busy, having a family crisis, or not responding to the invitations (Figure 1). Face-to-face surveys were conducted with 34 (57%) women and 26 (43%) of the women chose to be surveyed by telephone. The average age of the participating women was 33 years (standard deviation (SD) ± 4.5). Just under half of the women were primigravid and had a family history of diabetes (27, 45%), and two-thirds were classified as obese or overweight in early pregnancy (39, 65%) (Table 1). Most women were European (24, 40%) followed by Asian ethnicity (22, 37%). Women taking part were evenly distributed across the deprivation index: 18 (30%) women least deprived (levels 1–3) and 19 (32%) women (levels 4–6) and 22 (37%) women most deprived (levels 7–10) (Table 1). The demographics of the participating women are reflective of a cross section of the demographics of New Zealand's pregnant population [26–28] (Table 1).

Women were diagnosed with GDM at a mean of 27.8 ± 2.0-week gestation. At the time of the survey, participants had been checking their daily capillary blood glucose for an average of 6.8 ± 2.3 weeks (Table 1), with just over half checking their blood glucose four times a day (32, 53%) and the other participants six times a day (28, 47%). Ten women (17%) reported having a diagnosis of GDM from a previous pregnancy. Almost a third of women (18, 30%) were treated with diet alone; the remainder received a combination of dietary advice and medications. Thirteen (22%) women were treated with subcutaneous insulin for their GDM, 17 (28%) women metformin, and 12 (20%) women were treated with insulin and metformin (Table 1).

### 3.2. Views and Experiences about Achieving Recommended Glycaemic Treatment Targets

The majority of women correctly identified their glycaemic treatment targets (59, 98%) and viewed it as very important or important to try to adhere to these targets (Table 2). Documenting the blood glucose results were viewed as less important (56, 93%) compared to viewing adherence to the targets because women knew the results could be downloaded from the glucometer. These findings were similar across participants regardless of their glycaemic treatment targets (Table 2).

Almost two-thirds of women (37, 62%) described achieving their morning fasting glycaemic treatment target as most difficult. These findings were similar across participants, regardless of their recommended glycaemic targets (12 (60%) for less tight targets and 25 (62.5%) for tighter targets) (Table 2). The next most frequent difficulty reported for women to achieve their recommended glycaemic targets was after their evening meal (11, 18%). Again, these findings were similar across participants regardless of their glycaemic treatment targets (Table 2). Almost two-thirds of women (37, 62%) experienced being always or frequently hungry (Table 3).

### 3.3. Enablers to Achieving Optimal Blood Glucose Control

Participants were asked to identify what helped them when learning to test their capillary blood glucose levels. All 60 (100%) women indicated that having a health professional to demonstrate the collection of capillary blood glucose on themselves and then watch the participant perform it was helpful (Table 3). Fifty-six (93%) women opted to comment further about other factors that they felt were helpful for learning self-monitoring of blood glucose. These related mainly to group or individual teaching. Forty-four (79%) of the women who commented further stated that they found group sessions helpful with some women explaining that they enjoyed talking to other women and recognising that they are not alone living with GDM. A smaller proportion of women (12, 21%) received additional one-to-one teaching sessions and enjoyed them as these enabled them to ask “stupid” questions, they could ask the teacher to slow down when English was their second language, or they felt less as though it was “mass produced” and liked to be treated more as an individual. Over a third of women (22, 37%) identified Google as a helpful tool. It is unclear which websites they visited and in which language.

Support from family, friends, and work colleagues was seen as enabling for achieving glycaemic control. Over half of the women (33, 55%) indicated that they found it helpful to be asked about their capillary blood glucose levels and to be reminded to do them by their partners, children, extended family members, and work colleagues. Having their meals cooked by either their partners or extended family members, who incorporated the GDM diet recommendations, was found to be helpful for nearly half of the women (28, 47%) (Table 4). Comments indicated that this enabled women to eat more vegetables and stopped them from buying confectionary or sugar-sweetened beverages (fizzy drinks). Further comments around supportive provision of food by others included colleagues organising healthy morning teas at work and friends providing healthy food choices for baby showers. While nearly two-thirds of women (37, 62%) indicated that the cost associated with the GDM diagnosis, such as food, petrol, or child care, stayed the same, some women (8, 13%) reported reduced food costs since being diagnosed with GDM as an enabler due to buying fewer take-away meals (fast foods) (Table 3).

All women attended Diabetes in Pregnancy Services where they saw a range of health professionals. Most women (47, 78%) attended the clinic fortnightly (Table 4). Support from health professionals was valued. Over two-thirds of the women (41, 68%) appreciated that health professionals took time to listen and explain (Table 3). One (1.7%) woman could email the endocrinologist for advice and appreciated their prompt response.

### 3.4. Barriers to Achieving Optimal Blood Glucose Control

All women received written information about GDM, which explained the importance of healthy eating and its effect on blood glucose levels and how to self-monitor capillary blood glucose levels. Barriers to this written information included feeling overwhelmed with the amount of written material and not being able to read it in their first language. Women requested to receive visual information (16, 27%) rather than words for food choices, food label reading, how to perform the finger pricks for capillary blood glucose collection, and how to give subcutaneous insulin injections (Table 4). Over half of the women (33, 55%) found it difficult that the written information was in English and wanted the health information in their first language for themselves and for their families to better understand what GDM is and what optimal blood glucose control meant (Table 4). Hindi was the language most frequently requested (9, 27% women), followed by Samoan (6, 18% women) and then Chinese and Māori each by 5 (15%) women. This reflects the ethnic diversity of this cohort of women (Table 1).

Over a third of women (23, 38%) reported being offered unhealthy food by family, friends, and work colleagues and their lack of understanding as barriers to achieving optimal glycaemic control.

When engaging with the Diabetes in Pregnancy Services women, just over a fifth of women (13, 22%) reported a judgemental attitude by health professionals, being impatient with them, and being not believed that they had tried their hardest to stay within their recommended glycaemic treatment targets as a barrier. Inconsistent information by health professionals (10, 17%), never seeing the same health professional twice (8, 13%), and long waiting hours at the clinic (7, 12%) were also experienced as difficult (Table 4).

An increased cost for buying more vegetables, fresh fruits, and wholemeal bread was reported as a barrier by a quarter of women (15, 25%) (Table 4).

## 4. Discussion

In this survey, women with GDM identified enablers and barriers to achieving optimal glycaemic control. While achieving optimal glycaemic control was viewed as important, most women found it difficult to achieve their morning fasting glycaemic treatment targets, experienced hunger, and wanted the health information in their first language or visually displayed. For most women, food costs were not reported as a concern for the family budget. Being taught blood glucose testing in a group setting was considered helpful. Support from health professionals and family, friends, and work colleagues was valued. Barriers reported include long clinic waiting hours; inconsistent advice, judgemental attitudes, impatience, and not being believed by health professionals; and unhealthy food being offered by family members, friends, and work colleagues.

Health care providers recognise that teaching moments can be maximised by incorporating specific adult-learning principles and learning styles into their teaching strategies and providing written information that supports these learning styles [29]. The survey results showed that participants wished to be provided with better visual information and to have written information in their own language. Most women enjoyed group teaching sessions, although some preferred one-to-one sessions.

We found no published studies reporting on the effects of providing visual learning aids for women with gestational diabetes or the impact of having the information in their first language. One mixed-method study [30] among young people with type 1 diabetes (T1DM) in Norway found that a pictorial diary as a mobile phone app covering the topics diet, insulin dosage, physical activity, and pre- and postprandial glucose measurements all led to a change in the participants' applied knowledge about the management of their diabetes. This is an area requiring further research. For information to make sense and motivate behaviour change, it needs to be provided in a language best understood by the women with GDM [23–25, 31]. Women identified Google as a helpful tool. Health professionals need to be aware that women will access information beyond the clinic environment and the quality of this information may vary. Diabetes in Pregnancy Services should consider how they provide health information and the content of their teaching sessions. Health literacy providing clear and relevant health messages has been identified as an effective way to help people manage their own health care [31–34]. It would be challenging for Diabetes in Pregnancy Services to provide the information for women with GDM in all the languages identified through this survey. The solution may be to provide increased visual information which requires little language and/or translate the written information for the languages identified. The use of trained translators has been encouraged, as family members are often unfamiliar with the health care medical terms, may find it difficult talking about sensitive matters, and may have different degrees of English fluency [35].

Achieving adequate fasting blood glucose control prior to breakfast, also known as the dawn phenomenon [36], was identified as a challenge for most women in this survey, regardless of whether their recommended glycaemic targets were identified as less tight or tighter. In the literature, this has been identified previously for people with T1DM and T2DM [37] but we could not find any publications specifically relating to gestational diabetes. Anecdotal evidence through social media indicates that women with GDM do find this control difficult [38]. Various recommendations for achieving glycaemic control include subcutaneous insulin, walking after dinner, restricting carbohydrate intake at dinner time, late protein snack before bed time, and staying hydrated [39] but require further research for women with GDM. Two-thirds of women commented on being hungry. It is unclear from the survey if this relates to women trying to lower their morning fasting blood glucose with eating less at dinner time or eating very low carbohydrate meals. This would benefit from further exploration.

Some women identified barriers regarding health professional's attitude to not achieving adequate glycaemic control. These included judgemental attitude, not being believed when women stated that they were trying their hardest to follow all diet and pharmaceutical recommendations, and seeing a different health professional at each visit receiving inconsistent information. Findings from other qualitative studies reiterate these findings [31, 40, 41] and highlight the importance for health professional to have a woman-centred approach, not only focusing on blood glucose concentrations but investing time to listen, believing what the women say is true, and providing consistent information and continuity of care [41].

Support from family, friends, and work colleagues were appreciated by the women surveyed. These results are consistent with those of other studies [42, 43]. Barrier identification included unhealthy food being offered to them by family members, friends, and work colleagues, indicating a lack of understanding. Diabetes in Pregnancy Services may consider providing opportunity for family and friends to attend information sessions about GDM and its implication or include discussions about effective strategies for difficult situations at clinic appointments.

This study had some limitations. The participants were from two selected areas in New Zealand, and while they were a cross-sectional representation of the demographics of the New Zealand population, this did not include women living in rural or remote areas. The findings may not be generalised as different district health boards provide care for women with GDM through different models of care.

## 5. Conclusions

This survey identified barriers and enablers for women with GDM in achieving optimal glycaemic control from two different geographical locations in New Zealand. The results provide insights into women's views and experiences with GDM in achieving glycaemic control targets. Two-thirds of women found it difficult to achieve adequate fasting capillary blood glucose control, regardless of their recommended glycaemic targets, and identified the need for better strategies and adequate health professional and family support to manage this difficulty. Barriers for health information and literacy identified that health professionals need to consider using a women-centred and adult-learning style approach, provide visual aids, provide written information in relevant languages, and include extended family members when imparting knowledge on or teaching GDM-related skills. Long clinic waiting hours, inconsistent advice, judgemental attitudes, and not being believed by health professionals require further consideration when providing a health care service for women with GDM. Findings from this survey will be useful for developing strategies for Diabetes in Pregnancy Services to support women with GDM in achieving their glycaemic control.

## Figures and Tables

**Table 1:** Demographic characteristics of women who participated in the survey.

Characteristics	Women with less tight^1^ glycaemic treatment targets *n* = 20 (% or ±20)	Women with tighter^2^ glycaemic treatment targets *n* = 40 (% or ±40)	Women total *n* = 60 (% or ±60)
Age (years)^4^	34 (±4.3)	32 (±4.5)	33 (±4.5)
Primigravida (G_1_P_0_)^3^	9 (45)	18 (45)	27 (45)
*BMI category* ^5,3^			
Normal	8 (40)	13 (32.5)	21 (35)
Overweight	5 (25)	6 (15)	11 (18.3)
Obese (class I)	2 (10)	9 (22.5)	11 (18.3)
Obese (class II)	2 (10)	6 (15)	8 (13.3)
Obese (class II)	3 (15)	6 (15)	9 (15)
Total obese	7 (35)	21 (52.5)	28 (46.6)
*Ethnicity* ^6,3^			
European	12 (60)	12 (30)	24 (40)
Māori	—	6 (15)	6 (10)
Asian	7 (35)	15 (37.5)	22 (36.7)
Pacific Peoples	—	7 (17.5)	7 (11.6)
MELAA	1 (5)	—	1 (1.7)
*Highest educational qualifications after leaving school* ^7,3^			
(1) No qualification	1 (5)	2 (5)	3 (5)
(2) Level 1 certificate	—	2 (5)	2 (3.3)
(3) Level 2 certificate	2 (10)	2 (5)	4 (6.7)
(4) Level 3 certificate	2 (10)	4 (10)	6 (10)
(5) Level 4 certificate	—	4 (10)	4 (6.7)
(6) Level 5 and level 6 diploma	4 (20)	9 (22.5)	13 (21.7)
(7) Bachelor degree and level 7 qualification	8 (40)	17 (42.5)	25 (41.6)
(8) Postgraduate and honours degree	1 (5)	—	1 (1.7)
(9) Master's degree	2 (10)	—	2 (3.3)
*NZ deprivation index* ^8,3^			
1 (least deprived)	3 (15)	5 (12.5)	8 (13.5)
2	2 (10)	3 (7.5)	5 (8.4)
3	2 (10)	3 (7.5)	5 (8.4)
4	4 (20)	6 (15)	10 (16.7)
5	2 (10)	5 (12.5)	7 (11.8)
6	1 (5)	1 (1.7)	2 (3.4)
7	2 (10)	3 (7.5)	5 (8.5)
8	3 (15)	3 (7.5)	6 (10)
9	1 (5)	4 (10)	5 (8.7)
10 (most deprived)	—	6 (15)	6 (10)
*Lead maternity carer (LMC)* ^9,3^			
Midwife	19 (95)	36 (90)	55 (91.7)
Obstetrician	1 (5)	—	1 (1.7)
Hospital team	—	4 (10)	4 (6.7)
Gestational age at GDM diagnosis^4^ (weeks)	27.7 (±1.9)	27.9 (±2.0)	27.8 (±2.0)
Time of self-testing capillary blood glucose for (weeks)^4^	7.6 (±2.5)	6.4 (±2.1)	6.8 (±2.3)
Previous GDM^3^	4 (20)	6 (15)	10 (16.7)
Previous hypertension^3^	2 (10)	—	2 (3.3)
Current hypertension	—	3 (7.5)	3 (5)
Family history of hypertension^3^	8 (45)	16 (40)	24 (40)
Family history of diabetes^3^	7 (35)	20 (50)	27 (45)
Current smoker^3^	—	3 (7.5)	3 (15)
*Current treatment^3^*			
Diet only	7 (35)	11 (27.5)	18 (30)
Insulin and diet	2 (10)	11 (27.5)	13 (21.7)
Metformin and diet	5 (25)	12 (30)	17 (28.3)
Insulin, metformin, and diet	6 (30)	6 (15)	12 (20)

^1^Less tight glycaemic treatment targets for women with GDM: fasting blood glucose < 5.5 mmol/L, 1 hour postprandial < 8.0 mmol/L, and 2 hours postprandial < 7.0 mmol/L; ^2^tighter glycaemic treatment targets for women with GDM: fasting blood glucose ≤ 5.0 mmol/L, 1 hour postprandial ≤ 7.4 mmol/L, and 2 hours postprandial ≤6.7 mmol/L; ^3^figures are numbers and percentages; ^4^figures are mean and standard deviation; ^5^BMI categories: underweight < 18.50, normal range ≥ 18.55–24.99, overweight ≥ 25.00–29.99, obese (class I) ≥ 30.00–34.99, obese (class II)—severe obese ≥ 35.00–39.99 and obese (class II)—morbid obese ≥ 40.00 (according to WHO and Ministry of Health categories) [44, 45]; ^6^as categorised by New Zealand government statistics groups for major ethnic groups. MELAA is an acronym for Middle Eastern/Latin American/African (http://www.stats.govt.nz/Census/2013-census/profile-and-summary-reports/infographic-culture-identity.aspx); ^7^as categorised by New Zealand government statistics groups (http://www.stats.govt.nz/Census/2013-census/profile-and-summary-reports/qstats-education-training/highest-qualification.aspx); ^8^as categorised by New Zealand 2013 Deprivation Index, University of Otago, Department of Public Health. Deprivation score was unknown for one woman, as her address had no meshblock listed (http://www.otago.ac.nz/wellington/departments/publichealth/research/hirp/otago020194.html); ^9^a lead maternity carer (LMC) in New Zealand provides lead maternity care (is in charge). This can be either a midwife, obstetrician, or GP (https://www.midwife.org.nz/in-new-zealand/contexts-for-practice).

**Table 2:** Participants views and experiences of capillary blood glucose monitoring.

	Women with less tight glycaemic treatment targets *n* = 20 (% of 20)	Women with tighter glycaemic treatment targets *n* = 40 (% of 40)	Women total *n* = 60 (%)
Knew their glycaemic treatment targets	19 (98.3)	40 (100)	59 (98.3)
Viewed achieving glycaemic treatment targets as very important or important	20 (100)	39 (97.5)	59 (98.3)
Viewed documenting blood glucose results as very important or important	18 (90)	37 (92.5)	56 (93.3)
Experienced difficult fasting glycaemic treatment target (before breakfast)	12 (60)	25 (62.5)	37 (61.6)
Experienced difficult postprandial glycaemic treatment target (after dinner)	3 (15)	8 (20)	11 (18.3)

**Table 3:** Enablers identified by women with GDM^1^.

Enablers	Women with less tight glycaemic treatment targets *n* = 20 (% of 20)	Women with tighter glycaemic treatment targets *n* = 40 (% of 40)	Women total *N* = 60 (%)
Health professional demonstrating on themselves CBGT^2^	20 (100)	40 (100)	60 (100)
Watching participants perform CBGT^2^	20 (100)	40 (100)	60 (100)
Group teaching	11 (55)	33 (82.5)	44^3^ (78.5)
One to one teaching	6 (30)	6 (15)	12^3^ (21.4)
Health professionals listening and explaining	6 (30)	35 (87.5)	41 (68.3)
Being ask about their CBGC^4^ and reminded to do them	7 (35)	26 (65)	33 (55)
Others cooking incorporating GDM diet	11 (55)	17 (42.5)	28 (46.6)
Using Google	9 (45)	13 (32.5)	22 (36.6)
Going for walks/exercising together	6 (30)	9 (45)	15 (25)
Less costs	3 (15)	5 (12.5)	8 (13.3)

^1^Multiple answers were possible for this part of the survey; ^2^capillary blood glucose testing; ^3^results from 56 women; ^4^capillary blood glucose concentrations.

**Table 4:** Barriers identified by women with GDM.

Barriers	Women with less tight glycaemic treatment targets *n* = 20 (% of 20)	Women with tighter glycaemic treatment targets *n* = 40 (% of 40)	Women total *n* = 60 (%)
Health information available only in English	8 (40)	25 (62.5)	33 (55)
Health information in words not visual	5 (25)	11 (27.5)	16 (26.6)
Being offered unhealthy food by family, friends, and work colleagues	5 (25)	14 (35)	23 (38.3)
Impatient, not being believed, and being judged by health professionals	7 (35)	6 (15)	13 (21.6)
Inconsistent information by health professionals	4 (20)	6 (15)	10 (16.6)
Never seeing the same health professional twice	3 (15)	5 (12.5)	8 (13.3)
Long waiting hours at clinic	4 (20)	3 (7.5)	7 (11.6)
Being hungry	14 (70)	23 (57.5)	37 (61.6)
Increased costs	7 (35)	8 (20)	15 (25)
